# Novel Eurasian Highly Pathogenic Avian Influenza A H5 Viruses in Wild Birds, Washington, USA, 2014

**DOI:** 10.3201/eid2105.142020

**Published:** 2015-05

**Authors:** Hon S. Ip, Mia Kim Torchetti, Rocio Crespo, Paul Kohrs, Paul DeBruyn, Kristin G. Mansfield, Timothy Baszler, Lyndon Badcoe, Barbara Bodenstein, Valerie Shearn-Bochsler, Mary Lea Killian, Janice C. Pedersen, Nichole Hines, Thomas Gidlewski, Thomas DeLiberto, Jonathan M. Sleeman

**Affiliations:** US Geologic Survey–National Wildlife Health Center, Madison, Wisconsin, USA (H.S. Ip, B. Bodenstein, V. Shearn-Bochsler, J.M. Sleeman);; US Department of Agriculture, Ames, Iowa, USA (M.K. Torchetti, M.L. Killian, J.C. Pedersen, N. Hines);; Washington State University, Pullman, Washington, USA (R. Crespo, T. Baszler);; Washington Department of Agriculture, Olympia, Washington, USA (P. Kohrs, T. Baszler, L. Badcoe);; Washington Department of Fish and Wildlife, Olympia (P. DeBruyn, K.G. Mansfield);; US Department of Agriculture, Fort Collins, Colorado, USA (T. Gidlewski, T. DeLiberto)

**Keywords:** highly pathogenic avian influenza virus, HPAI, reassortment, A(H5N8), A(H5N2), H5N8, H5N2, intercontinental transmission, wild birds, United States, viruses, influenza

## Abstract

Novel Eurasian lineage avian influenza A(H5N8) virus has spread rapidly and globally since January 2014. In December 2014, H5N8 and reassortant H5N2 viruses were detected in wild birds in Washington, USA, and subsequently in backyard birds. When they infect commercial poultry, these highly pathogenic viruses pose substantial trade issues.

The novel Eurasian lineage clade 2.3.4.4 highly pathogenic avian influenza (HPAI) A(H5N8) virus (http://www.who.int/influenza/gisrs_laboratory/h5_nomenclature_clade2344/en/) spread rapidly and globally during 2014, substantially affecting poultry populations. The first outbreaks were reported during January 2014 in chickens and domestic ducks in South Korea and subsequently in China and Japan (*1**–**4*), reaching Germany, the Netherlands, and the United Kingdom by November 2014 and Italy in early December 2014 (*5*). Also in November 2014, a novel HPAI H5N2 virus was reported in outbreaks on chicken and turkey farms in Fraser Valley, British Columbia, Canada (*5*). This H5N2 influenza virus is a reassortant that contains the Eurasian clade 2.3.4.4 H5 plus 4 other Eurasian genes (polymerase acidic protein subunit, matrix protein, polymerase basic protein subunit [PB] 2, nonstructural protein) and 3 North American wild bird lineage genes (neuraminidase [NA], nucleoprotein, PB1) (*5*). Taiwan has recently reported novel reassortants of the H5 clade 2.3.4.4 with other Eurasian viruses (H5N2, H5N3).

The appearance of highly similar Eurasian H5N8 viruses in Asia, Europe, and now the United States suggests that this novel reassortant may be well adapted to certain waterfowl species, enabling it to survive long migrations (*6*). These appearances also represent a major change in Eurasian H5 virus circulation. After the reported spread of HPAI H5N1 virus in Asia, a large, interagency avian influenza virus (AIV) surveillance effort was implemented throughout the United States during April 2006–March 2011 (*7*). Of nearly 500,000 wild bird samples tested, none harbored Eurasian subtype H5 AIV. The overall prevalence of AIV was ≈11%, and most viruses (86%) were detected in dabbling ducks (family Anatidae) (*8*). Although H5N8 subtype viruses have been detected previously in the United States, all have been low pathogenicity AIV of North American wild bird lineage.

## Case Reports

After the November 2014 report of H5N2 HPAI outbreaks among poultry in British Columbia, the US Departments of Agriculture and Interior, together with state agency personnel, increased surveillance of poultry flocks, hunter-harvested wild birds, and wild bird die-offs along the US–Canada border. A wild bird die-off was reported on December 1, 2014, at Wiser Lake (48.9039N, 122.4799W) in Whatcom County, Washington, USA. The lake, which has a history of waterfowl deaths caused by lead poisoning and aspergillosis, is located ≈32 km from the location of the index cases in Fraser Valley. Up to 10,000 waterfowl were on the lake when the deaths began. The dead birds consisted primarily of mallards (*Anas platyrhynchos*), American wigeon (*A. americana*), and northern pintail (*A. acuta*), along with smaller numbers of other waterfowl species. 

Nine carcasses were submitted to the National Wildlife Health Center (Table 1); 6 were examined in detail. *Aspergillus fumigatus* was isolated from 5 birds with characteristic lesions of airsacculitis (Table 1). In addition, cloacal and/or oral swab samples from 5 birds had molecular assay results positive for influenza A and H5 (Table 1). A Eurasian lineage H5 clade 2.3.4.4 AIV, A/northern pintail/Washington/40964/2014 (H5N2) (GenBank taxon no. 1589662), was isolated from a lung specimen. Whole-genome sequencing indicated the virus was highly similar to the H5N2 reassortant virus from Canada. Both viruses have 3 RNA segments of North American wild bird lineage (PB1, nucleoprotein, and NA) and 5 RNA segments (PB2, polymerase acidic, HA, matrix protein, and nonstructural protein) that showed >99% similarity to 2014 Eurasian clade 2.3.4.4 H5N8 viruses (Table 2). According to World Organisation for Animal Health guidelines (*9*), the virus was consistent with HPAI on the basis of the amino acid sequence at the hemagglutinin cleavage site and in vivo assay results (intravenous pathogenicity index 2.57).

**Table 1:** Summary of influenza test results for samples from 10 birds from Washington, USA, 2014*

Sample ID no.	Species, common name	Necropsy	AIV status	Diagnostic finding
26080–001	Northern shoveler	Yes	PCR negative, isolation negative	Aspergillosis
26080–002	Northern pintail	Yes	Subtype H5N2	Aspergillosis, HPAI
26080–003	American wigeon	Yes	PCR positive, isolation negative	Aspergillosis
26080–004	American wigeon	No	PCR negative, isolation negative	ND
26080–005	American wigeon	No	PCR negative, isolation negative	ND
26080–006†	Mallard	Yes	PCR positive, isolation negative	Aspergillosis
26080–007†	Mallard	Yes	PCR positive, isolation negative	Aspergillosis
26080–008†	Mallard	No	PCR positive, isolation negative	ND
26080–009	Trumpeter swan	Yes	PCR negative, isolation negative	Emaciation
RW099878	Gyrfalcon	Yes	Subtype H5N8	HPAI

*AIV, avian influenza virus; HPAI, highly pathogenic avian influenza; ID, identification, ND, not determined. †Specimens tested by the Washington Animal Disease Diagnostic Laboratory.

**Table 2:** Nucleotide identity between the influenza A(H5N2) and A(H5N8) viruses from Washington, USA, and their nearest homologues in GenBank as of January 8, 2015*

Virus from Washington State	Nearest homologue	% Identity
A/Northern pintail/Washington/40964/2014 (H5N2)		
PB2	A/bean goose/Korea/H40/2014	99.5
PB1	A/bufflehead/California/3118/2011	99.0
PA	A/common teal/Korea/H455–30/2014	99.5
HA	A/crane/Kagoshima/KU1/2014	99.3
NP	A/American green-winged teal/Ohio/13OS2084/2013	99.1
NA	A/bufflehead/California/4935/2012	99.0
MP	A/Baikal teal/Korea/S005/2014	100.0
NS	A/Baikal teal/Korea/Donglim3/2014	99.9
A/gyrfalcon/Washington/40188–6/2014 (H5N8)		
PB2	A/bean goose/Korea/H40/2014	99.6
PB1	A/Baikal teal/Korea/H41/2014	99.4
PA	A/Baikal teal/Korea/Donglim3/2014	99.3
HA	A/crane/Kagoshima/KU1/2014	99.2
NP	A/Baikal teal/Korea/H41/2014	99.5
NA	A/Coot/Korea/H81/2014	99.4
MP	A/Baikal teal/Korea/Donglim3/2014	99.9
NS	A/Baikal teal/Korea/Donglim3/2014	100.0

*HA, hemagglutinin; MP, matrix protein; NA, neuraminidase; NP, nucleoprotein; NS, nonstructural, PA, polymerase acidic; PB1 and 2, polymerase basic 1 and 2.

In a related event, on December 6, 2014, an American wigeon was captured and partially consumed by a captive-reared gyrfalcon (*Falco rusticolus*) in Whatcom County, ≈8 km from Wiser Lake. The wigeon remains were also fed to 3 other gyrfalcon and gyrfalcon–peregrine hybrids at a farm with another 25 raptors and 40 pigeons. The first falcon (RW099878) died on December 8 and was submitted to the National Wildlife Health Center. The second falcon (RX085847) also died on December 8 and the third (RX093091) on December 11; the fourth (RX084955) was euthanized on December 11. Carcasses of these last 3 falcons were submitted to the Washington Animal Disease Diagnostic Laboratory. No further deaths or illnesses have been reported among other raptors at the facility. Histologic and pathologic findings for the 3 raptors were consistent with those described in previous reports of H5N8 infections (*1**,**6*), and the severity of the lesions corresponded to virus concentrations detected in the tissues by molecular assays; results will be further detailed in a subsequent publication.

Molecular assay results for oral and cloacal swab samples and major organ and brain samples from falcon RW099878 were positive for influenza A and H5 viruses. For falcons RW099878 and RX085847, partial HA and NA genes were directly sequenced from brain and oral swab samples. A Eurasian lineage H5 clade 2.3.4.4 AIV, A/gyrfalcon/Washington/41088-6/2014 (H5N8) (GenBank taxon no. 1589663), was isolated from the brain of falcon RW099878. All 8 RNA segments for the strain were >99% similar to those for 2014 Group A H5N8 strains from South Korea (*3*) (Table 2); the amino acid sequence at the hemagglutinin cleavage site and in vivo assay results (intravenous pathogenicity index 2.65) were consistent with HPAI.

**Figure 1:**
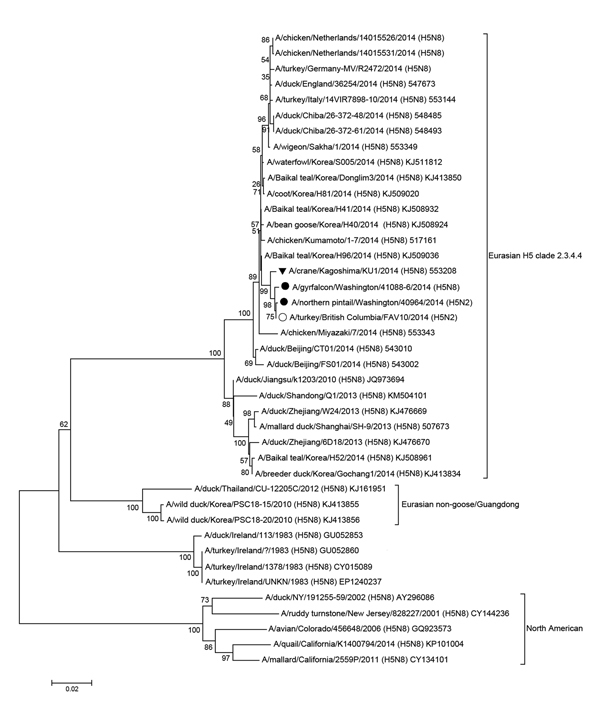
Phylogenetic comparison of the complete hemagglutinin genes of highly pathogenic avian influenza A(H5N2) and A(H5N8) strains from the United States with strains from Asia, Europe, and Canada. Solid circles indicate H5N2 and H5N8 strains from the United States; open circle indicates H5N2 strain from Canada; black triangle indicates H5N8 strain from a crane in Japan. Sequences were aligned by using MUSCLE, and phylogenetic and molecular evolutionary analyses were conducted by using MEGA version 5, using the neighbor-joining tree-building method, with 1,000 bootstrap replicates (*10*). Scale bar indicates nucleotide substitutions per site. Analysis was done with viruses that were phylogenetically representative of appropriate lineages (Influenza Virus Resource Database, http://www.ncbi.nlm.nih.gov/genomes/FLU/FLU.html).

**Figure 2:**
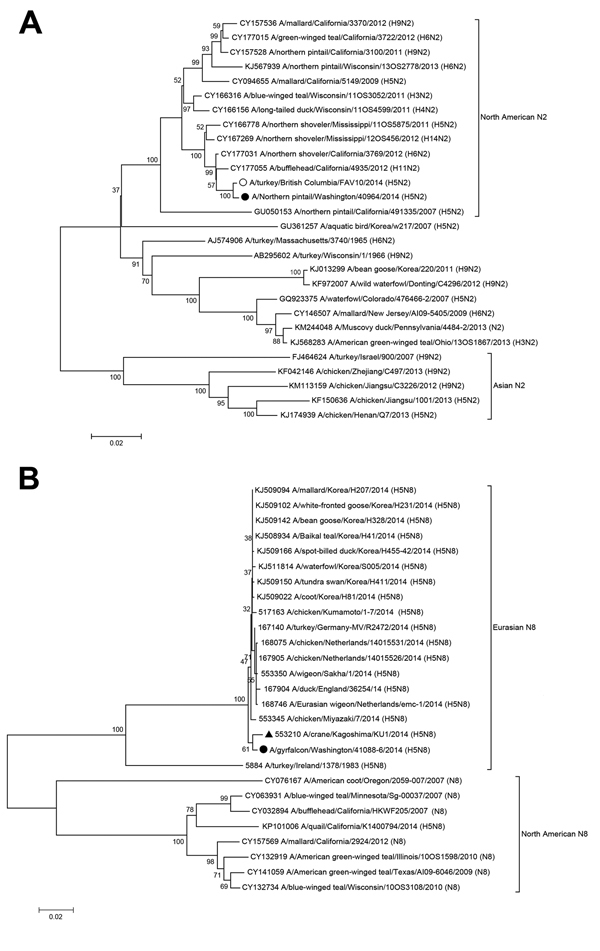
Phylogenetic comparison of the complete neuraminidase genes of highly pathogenic avian influenza A(H5N2) (panel A) and A(H5N8) (panel B) strains from the United States with strains from Asia, Europe, and Canada. Solid circles indicate H5N2 and H5N8 strains from the United States; black triangle indicates H5N8 strain from a crane in Japan. Sequences were aligned by using MUSCLE, and phylogenetic and molecular evolutionary analyses were conducted by using MEGA version 5, using the neighbor-joining tree-building method, with 1,000 bootstrap replicates (*10*). Scale bar indicates nucleotide substitutions per site. Analysis was done with viruses that were phylogenetically representative of appropriate lineages (Influenza Virus Resource Database, http://www.ncbi.nlm.nih.gov/genomes/FLU/FLU.html).

Phylogenetic analysis of the H5 clade 2.3.4.4 viruses detected in the United States resulted in 3 major findings (Figures 1, 2; Technical Appendix). First, the Eurasian lineage avian H5N8 clade 2.3.4.4 virus survived introduction into North America in its entirety. Second, introduction of Eurasian H5N8 virus into North America appears to be independent from introductions of the virus into Europe. Third, the duration of circulation of H5N8 virus in the Pacific flyway (California, Idaho, Nevada, Oregon, Utah, and Washington, USA) is unknown, but it was sufficient for reassortment with low pathogenicity North American lineage wild bird AIV (Figure 1).

## Conclusions

The ongoing circulation of these Eurasian HPAI H5 viruses in wild birds considerably alters the potential risks and subsequent consequences for US poultry and wildlife rehabilitation centers. Detection of HPAI H5N8 virus in apparently healthy common teal (*A. crecca*), Eurasian wigeon (*A. penelope*), mallard, spot-billed duck (*A. poecilorhyncha*), and tundra swans (*C. columbianus*) (*3**,**5*) suggests that wild birds may contribute to further spread of this HPAI H5 lineage in North America. However, culling and otherwise disturbing wild birds or their habitats has not been shown to be beneficial in the control of avian influenza (*11*). The scientifically supported management action (*11*) is to enhance biosecurity to minimize contacts between poultry, wild birds, and their fomites (*11*). In addition, hunters should be cognizant of risks from handling potentially infected carcasses (http://www.aphis.usda.gov/animal_health/birdbiosecurity/downloads/USDA_HntrCd_Hi.pdf).

Examination of wild bird surveillance samples collected before December 2014 may provide further insight into the timing and route of introduction of the Eurasian clade 2.3.4.4 H5N8 virus into North America. In addition, enhanced and ongoing influenza surveillance in wild birds and poultry will contribute to a better understanding of the geographic distribution and species involved in the spread of these HPAI H5 viruses. Together, these data may enable waterfowl managers and poultry producers to better assess and manage disease risks. Human infections have not been associated with either virus; however, H5 clade 2.3.4 H5N1 virus has caused human death, so caution is warranted. During preparation of this article, H5N8 was reported in wild birds and poultry along the Pacific flyway; novel H5N2 virus was detected in Idaho, Oregon, and Washington; and another novel reassortant H5N1 was detected in Washington and British Columbia (*5*). These detections have had major effects on US poultry trade (*12*).

Technical Appendix Phylogenetic analysis of the 6 internal genes of the influenza A(H5N2) and A(H5N8) viruses.
