# Supplementary material for: Novel Eurasian Highly Pathogenic Avian Influenza A H5 Viruses in Wild Birds, Washington, USA, 2014
Source: Emerg Infect Dis. 2015 May;21(5):886–90. doi: 10.3201/eid2105.142020 (PMC4412248; doi:10.3201/eid2105.142020)
Supplement: Technical Appendix — Phylogenetic analysis of the 6 internal genes of the influenza A(H5N2) and A(H5N8) viruses. [file 14-2020-Techapp-s1.pdf]

Article DOI: http://dx.doi.org/10.3201/eid2105.142020

Novel Eurasian Highly Pathogenic Influenza A(H5) Viruses in Wild Birds, Washington State, USA, 2014

Technical Appendix

A

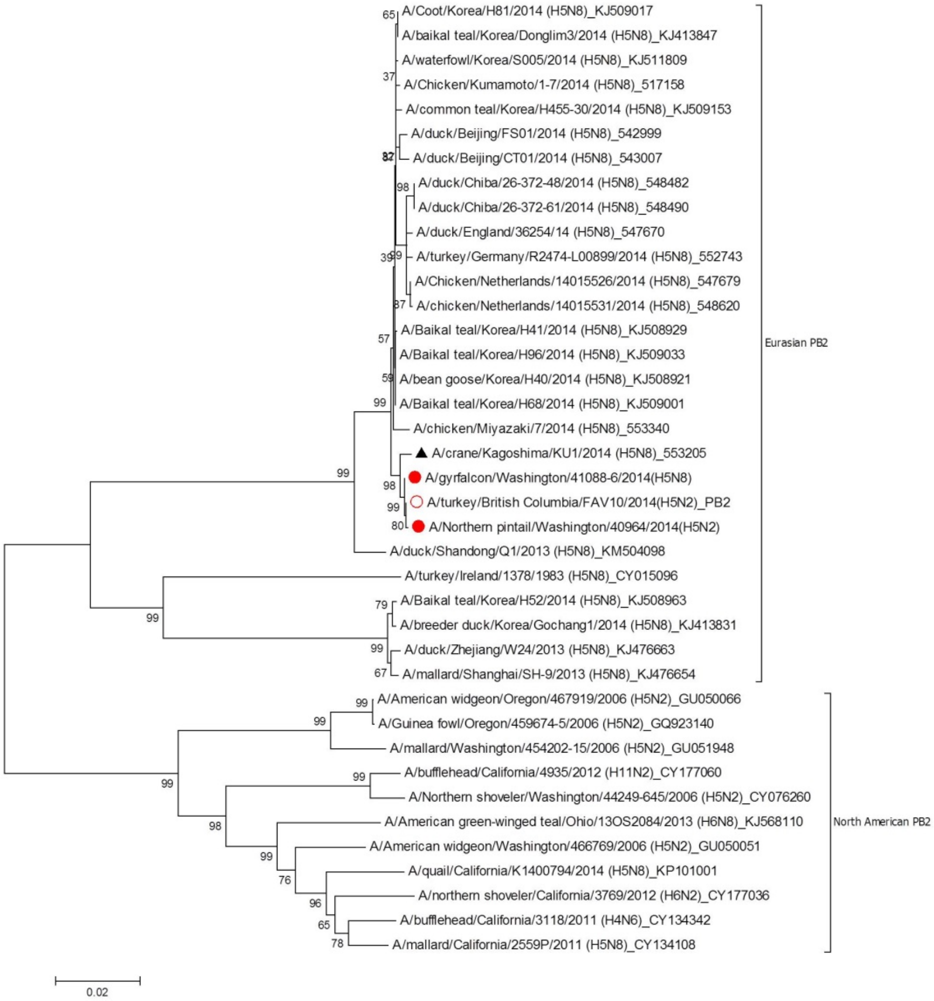

B

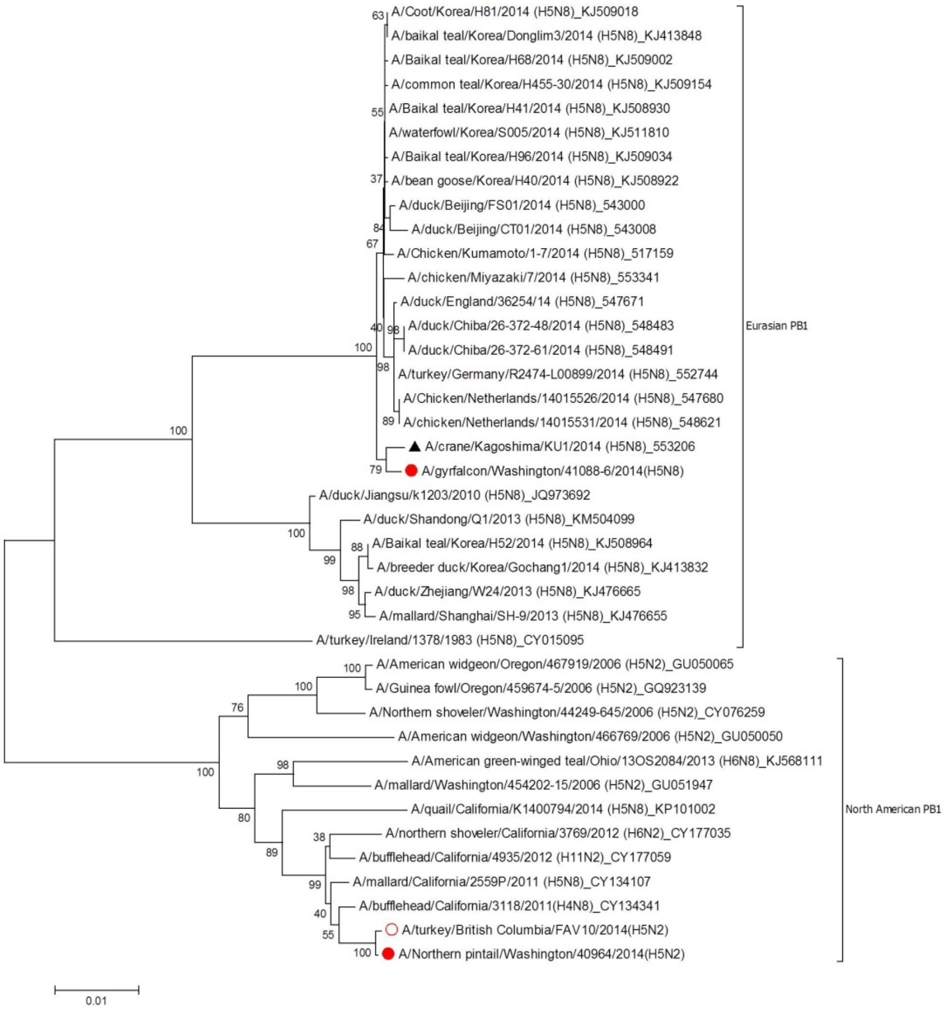
A/Coot/Korea/H81/2014 (H5N8)_KJ509018
A/baikal teal/Korea/Donglim3/2014 (H5N8)_KJ413848
A/Baikal teal/Korea/H68/2014 (H5N8)_KJ509002
A/common teal/Korea/H455-30/2014 (H5N8)_KJ509154
A/Baikal teal/Korea/H41/2014 (H5N8)_KJ508930
A/waterfowl/Korea/S005/2014 (H5N8)_KJ511810
A/Baikal teal/Korea/H96/2014 (H5N8)_KJ509034
A/bean goose/Korea/H40/2014 (H5N8)_KJ508922
A/duck/Beijing/FS01/2014 (H5N8)_543000
A/duck/Beijing/CT01/2014 (H5N8)_543008
A/Chicken/Kumamoto/1-7/2014 (H5N8)_517159
A/chicken/Miyazaki/7/2014 (H5N8)_553341
A/duck/England/36254/14 (H5N8)_547671
A/duck/Chiba/26-372-48/2014 (H5N8)_548483
A/duck/Chiba/26-372-61/2014 (H5N8)_548491
A/turkey/Germany/R2474-L00899/2014 (H5N8)_552744
A/Chicken/Netherlands/14015526/2014 (H5N8)_547680
A/chicken/Netherlands/14015531/2014 (H5N8)_548621
A/crane/Kagoshima/KU1/2014 (H5N8)_553206
A/gyrfalcon/Washington/41088-6/2014(H5N8)
A/duck/Jiangsu/k1203/2010 (H5N8)_JQ973692
A/duck/Shandong/Q1/2013 (H5N8)_KM504099
A/Baikal teal/Korea/H52/2014 (H5N8)_KJ508964
A/breeder duck/Korea/Gochang1/2014 (H5N8)_KJ413832
A/duck/Zhejiang/W24/2013 (H5N8)_KJ476665
A/mallard/Shanghai/SH-9/2013 (H5N8)_KJ476655
A/turkey/Ireland/1378/1983 (H5N8)_CY015095
Eurasian PB1
A/American widgeon/Oregon/467919/2006 (H5N2)_GU050065
A/Guinea fowl/Oregon/459674-5/2006 (H5N2)_GQ923139
A/Northern shoveler/Washington/44249-645/2006 (H5N2)_CY076259
A/American widgeon/Washington/466769/2006 (H5N2)_GU050050
A/American green-winged teal/Ohio/13OS2084/2013 (H6N8)_KJ568111
A/mallard/Washington/454202-15/2006 (H5N2)_GU051947
A/quail/California/K1400794/2014 (H5N8)_KP101002
A/northern shoveler/California/3769/2012 (H6N2)_CY177035
A/bufflehead/California/4935/2012 (H11N2)_CY177059
A/mallard/California/2559P/2011 (H5N8)_CY134107
A/bufflehead/California/3118/2011(H4N8)_CY134341
A/turkey/British Columbia/FAV10/2014(H5N2)
A/Northern pintail/Washington/40964/2014(H5N2)
North American PB1
0.01

C

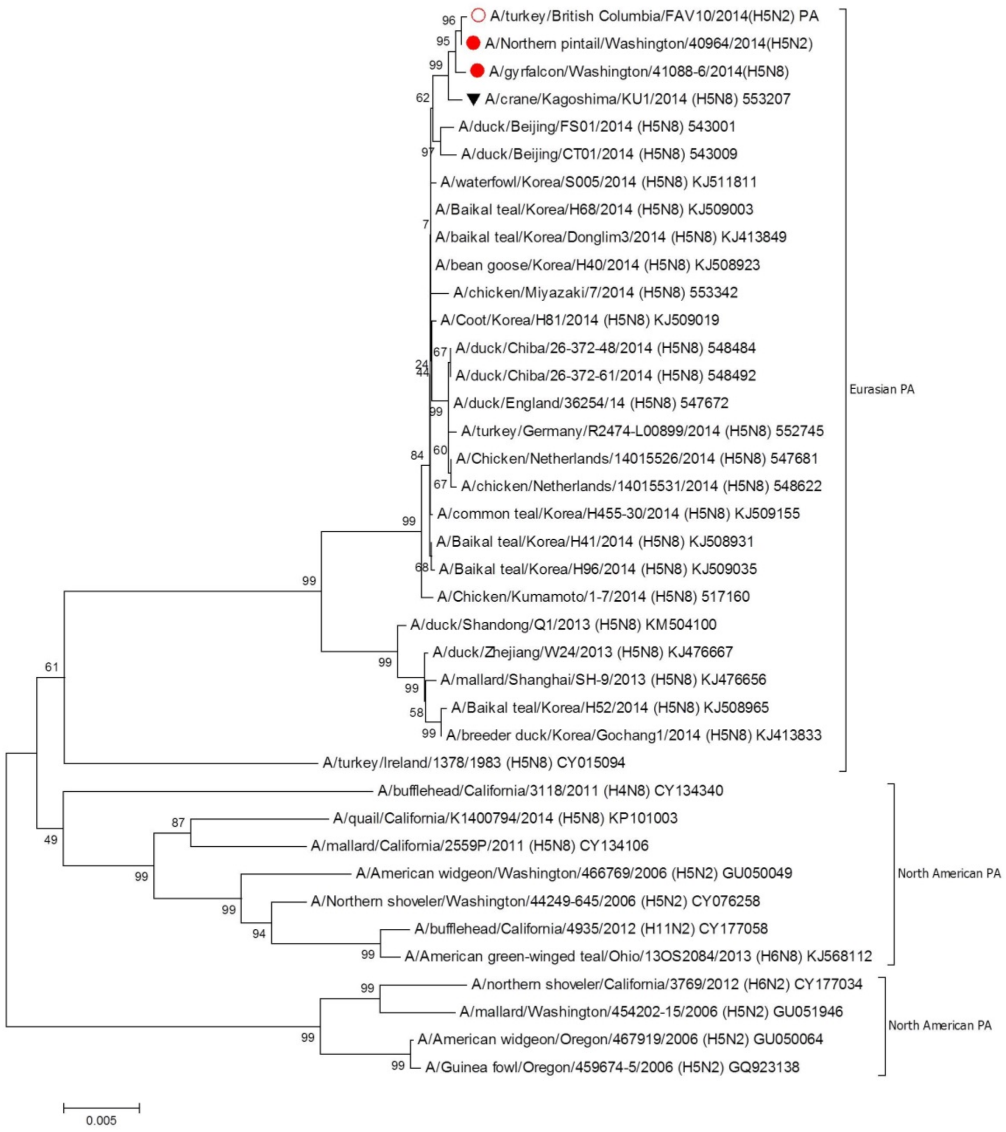
A/turkey/British Columbia/FAV10/2014(H5N2) PA
A/Northern pintail/Washington/40964/2014(H5N2)
A/gyrfalcon/Washington/41088-6/2014(H5N8)
A/crane/Kagoshima/KU1/2014 (H5N8) 553207
A/duck/Beijing/FS01/2014 (H5N8) 543001
A/duck/Beijing/CT01/2014 (H5N8) 543009
A/waterfowl/Korea/S005/2014 (H5N8) KJ511811
A/Baikal teal/Korea/H68/2014 (H5N8) KJ509003
A/baikal teal/Korea/Donglim3/2014 (H5N8) KJ413849
A/bean goose/Korea/H40/2014 (H5N8) KJ508923
A/chicken/Miyazaki/7/2014 (H5N8) 553342
A/Coot/Korea/H81/2014 (H5N8) KJ509019
A/duck/Chiba/26-372-48/2014 (H5N8) 548484
A/duck/Chiba/26-372-61/2014 (H5N8) 548492
A/duck/England/36254/14 (H5N8) 547672
A/turkey/Germany/R2474-L00899/2014 (H5N8) 552745
A/Chicken/Netherlands/14015526/2014 (H5N8) 547681
A/chicken/Netherlands/14015531/2014 (H5N8) 548622
A/common teal/Korea/H455-30/2014 (H5N8) KJ509155
A/Baikal teal/Korea/H41/2014 (H5N8) KJ508931
A/Baikal teal/Korea/H96/2014 (H5N8) KJ509035
A/Chicken/Kumamoto/1-7/2014 (H5N8) 517160
A/duck/Shandong/Q1/2013 (H5N8) KM504100
A/duck/Zhejiang/W24/2013 (H5N8) KJ476667
A/mallard/Shanghai/SH-9/2013 (H5N8) KJ476656
A/Baikal teal/Korea/H52/2014 (H5N8) KJ508965
A/breeder duck/Korea/Gochang1/2014 (H5N8) KJ413833
A/turkey/Ireland/1378/1983 (H5N8) CY015094
Eurasian PA
A/bufflehead/California/3118/2011 (H4N8) CY134340
A/quail/California/K1400794/2014 (H5N8) KP101003
A/mallard/California/2559P/2011 (H5N8) CY134106
A/American widgeon/Washington/466769/2006 (H5N2) GU050049
A/Northern shoveler/Washington/44249-645/2006 (H5N2) CY076258
A/bufflehead/California/4935/2012 (H11N2) CY177058
A/American green-winged teal/Ohio/13OS2084/2013 (H6N8) KJ568112
North American PA
A/northern shoveler/California/3769/2012 (H6N2) CY177034
A/mallard/Washington/454202-15/2006 (H5N2) GU051946
A/American widgeon/Oregon/467919/2006 (H5N2) GU050064
A/Guinea fowl/Oregon/459674-5/2006 (H5N2) GQ923138
North American PA
0.005

D

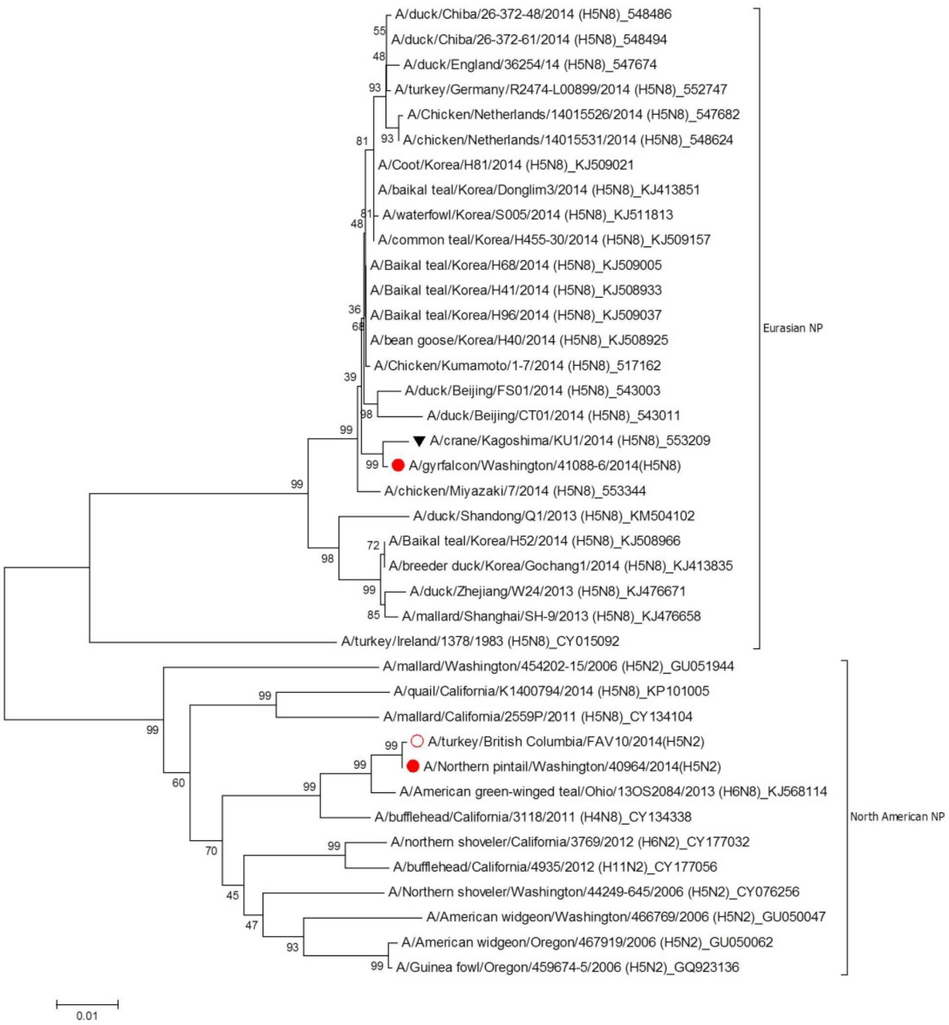

A/duck/Chiba/26-372-48/2014 (H5N8)_548486
A/duck/Chiba/26-372-61/2014 (H5N8)_548494
A/duck/England/36254/14 (H5N8)_547674
A/turkey/Germany/R2474-L00899/2014 (H5N8)_552747
A/Chicken/Netherlands/14015526/2014 (H5N8)_547682
A/chicken/Netherlands/14015531/2014 (H5N8)_548624
A/Coot/Korea/H81/2014 (H5N8)_KJ509021
A/baikal teal/Korea/Donglim3/2014 (H5N8)_KJ413851
A/waterfowl/Korea/S005/2014 (H5N8)_KJ511813
A/common teal/Korea/H455-30/2014 (H5N8)_KJ509157
A/Baikal teal/Korea/H68/2014 (H5N8)_KJ509005
A/Baikal teal/Korea/H41/2014 (H5N8)_KJ508933
A/Baikal teal/Korea/H96/2014 (H5N8)_KJ509037
A/bean goose/Korea/H40/2014 (H5N8)_KJ508925
A/Chicken/Kumamoto/1-7/2014 (H5N8)_517162
A/duck/Beijing/FS01/2014 (H5N8)_543003
A/duck/Beijing/CT01/2014 (H5N8)_543011
A/crane/Kagoshima/KU1/2014 (H5N8)_553209
A/gyrfalcon/Washington/41088-6/2014(H5N8)
A/chicken/Miyazaki/7/2014 (H5N8)_553344
A/duck/Shandong/Q1/2013 (H5N8)_KM504102
A/Baikal teal/Korea/H52/2014 (H5N8)_KJ508966
A/breeder duck/Korea/Gochang1/2014 (H5N8)_KJ413835
A/duck/Zhejiang/W24/2013 (H5N8)_KJ476671
A/mallard/Shanghai/SH-9/2013 (H5N8)_KJ476658
A/turkey/Ireland/1378/1983 (H5N8)_CY015092
Eurasian NP
A/mallard/Washington/454202-15/2006 (H5N2)_GU051944
A/quail/California/K1400794/2014 (H5N8)_KP101005
A/mallard/California/2559P/2011 (H5N8)_CY134104
A/turkey/British Columbia/FAV10/2014(H5N2)
A/Northern pintail/Washington/40964/2014(H5N2)
A/American green-winged teal/Ohio/13OS2084/2013 (H6N8)_KJ568114
A/bufflehead/California/3118/2011 (H4N8)_CY134338
A/northern shoveler/California/3769/2012 (H6N2)_CY177032
A/bufflehead/California/4935/2012 (H11N2)_CY177056
A/Northern shoveler/Washington/44249-645/2006 (H5N2)_CY076256
A/American widgeon/Washington/466769/2006 (H5N2)_GU050047
A/American widgeon/Oregon/467919/2006 (H5N2)_GU050062
A/Guinea fowl/Oregon/459674-5/2006 (H5N2)_GQ923136
North American NP
0.01

E

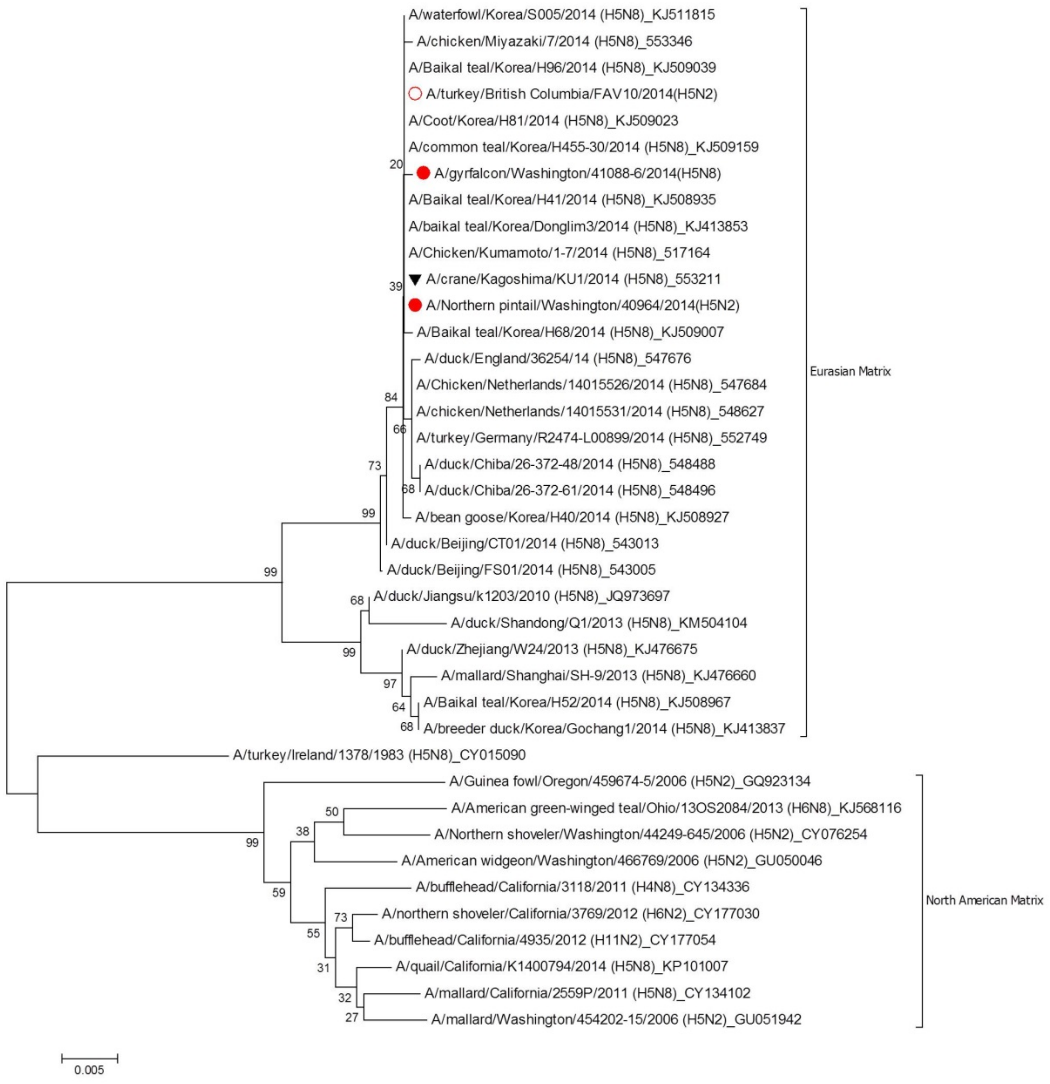

A/waterfowl/Korea/S005/2014 (H5N8)_KJ511815
A/chicken/Miyazaki/7/2014 (H5N8)_553346
A/Baikal teal/Korea/H96/2014 (H5N8)_KJ509039
A/turkey/British Columbia/FAV10/2014(H5N2)
A/Coot/Korea/H81/2014 (H5N8)_KJ509023
A/common teal/Korea/H455-30/2014 (H5N8)_KJ509159
A/gyrfalcon/Washington/41088-6/2014(H5N8)
A/Baikal teal/Korea/H41/2014 (H5N8)_KJ508935
A/baikal teal/Korea/Donglim3/2014 (H5N8)_KJ413853
A/Chicken/Kumamoto/1-7/2014 (H5N8)_517164
A/crane/Kagoshima/KU1/2014 (H5N8)_553211
A/Northern pintail/Washington/40964/2014(H5N2)
A/Baikal teal/Korea/H68/2014 (H5N8)_KJ509007
A/duck/England/36254/14 (H5N8)_547676
A/Chicken/Netherlands/14015526/2014 (H5N8)_547684
A/chicken/Netherlands/14015531/2014 (H5N8)_548627
A/turkey/Germany/R2474-L00899/2014 (H5N8)_552749
A/duck/Chiba/26-372-48/2014 (H5N8)_548488
A/duck/Chiba/26-372-61/2014 (H5N8)_548496
A/bean goose/Korea/H40/2014 (H5N8)_KJ508927
A/duck/Beijing/CT01/2014 (H5N8)_543013
A/duck/Beijing/FS01/2014 (H5N8)_543005
A/duck/Jiangsu/k1203/2010 (H5N8)_JQ973697
A/duck/Shandong/Q1/2013 (H5N8)_KM504104
A/duck/Zhejiang/W24/2013 (H5N8)_KJ476675
A/mallard/Shanghai/SH-9/2013 (H5N8)_KJ476660
A/Baikal teal/Korea/H52/2014 (H5N8)_KJ508967
A/breeder duck/Korea/Gochang1/2014 (H5N8)_KJ413837
Eurasian Matrix
A/turkey/Ireland/1378/1983 (H5N8)_CY015090
A/Guinea fowl/Oregon/459674-5/2006 (H5N2)_GQ923134
A/American green-winged teal/Ohio/13OS2084/2013 (H6N8)_KJ568116
A/Northern shoveler/Washington/44249-645/2006 (H5N2)_CY076254
A/American widgeon/Washington/466769/2006 (H5N2)_GU050046
A/bufflehead/California/3118/2011 (H4N8)_CY134336
A/northern shoveler/California/3769/2012 (H6N2)_CY177030
A/bufflehead/California/4935/2012 (H11N2)_CY177054
A/quail/California/K1400794/2014 (H5N8)_KP101007
A/mallard/California/2559P/2011 (H5N8)_CY134102
A/mallard/Washington/454202-15/2006 (H5N2)_GU051942
North American Matrix
20
39
84
66
73
68
99
99
68
99
97
64
68
50
38
99
59
73
55
31
32
27
0.005

F

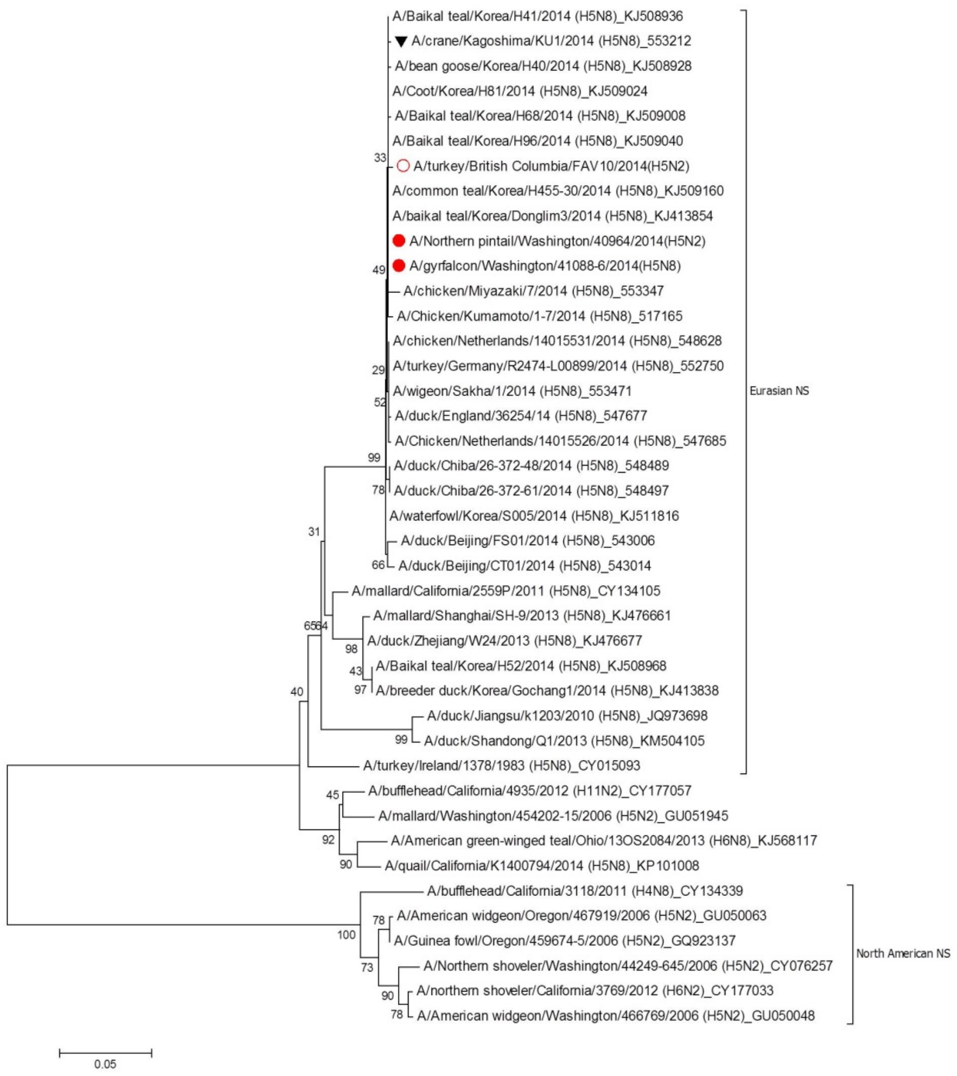

Technical Appendix Figure, panels A–F. Phylogenetic analysis of the 6 internal genes of the influenza A(H5N2) and (H5N8) viruses: A, polymerase basic (PB) 2; B, PB1; C, polymerase acidic (PA); D, nucleoprotein (NP); E, matrix (M); and F, nonstructural (NS). Red circles indicate highly pathogenic avian influenza (HPAI) subtype H5N2 and H5N8 strains from the United States; open red circle indicates HPAI subtype H5N2 virus from Canada; black triangle represents HPAI subtype H5N8 derived from A/crane/Kagoshima/KU1/2014(H5N8) in Japan. Sequences were aligned by using MUSCLE, and phylogenetic and molecular evolutionary analyses were conducted by using MEGA version 5, using the neighbor-joining tree-building method, with 1,000 bootstrap replicates (*1*). Analysis was done with viruses that were phylogenetically representative of appropriate lineages (Influenza Virus Resource database, http://www.ncbi.nlm.nih.gov/genomes/FLU/FLU.html).

Reference

<jrn>1. Tamura K, Peterson D, Peterson N, Stecher G, Nei M, Kumar S. MEGA5: Molecular Evolutionary Genetics Analysis using Maximum Likelihood, Evolutionary Distance, and Maximum Parsimony Methods. Mol Biol Evol. 2011;28:2731–9. PubMed http://dx.doi.org/10.1093/molbev/msr121</jrn>
